# A novel inflammation-related prognostic biomarker for predicting the disease-free survival of patients with colorectal cancer

**DOI:** 10.1186/s12957-022-02550-0

**Published:** 2022-03-11

**Authors:** Xiaoling Cai, Fa Chen, Lisheng Liang, Weizhong Jiang, Xing Liu, Dong Wang, Yunli Wu, Jinyan Chen, Guoxian Guan, Xian-e Peng

**Affiliations:** 1 Department of Epidemiology and Health Statistics, Fujian Provincial Key Laboratory of Environment Factors and Cancer, The School of Public Health, Fujian Medical University, Fuzhou, China; 2 Department of Nursing, Fujian Medical University Union Hospital, Fuzhou, China; 3 Department of Colorectal Surgery, Fujian Medical University Union Hospital, Fuzhou, China; 4 Department of Colorectal Surgery, The First Affiliated Hospital of Fujian Medical University, Fuzhou, China; 5 Department of General Surgery, Fujian Medical University Union Hospital, Fuzhou, China; 6 Key Laboratory of Ministry of Education for Gastrointestinal Cancer, Fujian Medical University, Fuzhou, China; 7 Institute for Immunology, Fujian Academy of Medical Sciences, Fuzhou, Fujian China

**Keywords:** Colorectal cancer, Inflammatory biomarkers, Disease-free survival, Prognosis, Decision curve analysis

## Abstract

**Background:**

To develop and evaluate the prognostic value of a comprehensive inflammatory biomarker for postoperative colorectal cancer (CRC) patients.

**Methods:**

A total of 646 CRC patients were recruited between August 2017 and December 2019 from Fujian Medical University Union Hospital, with follow-up data up to 2021. The least absolute shrinkage and selection operator method (LASSO) was used to select inflammation indicators in order to construct a comprehensive biomarker (named NSAP). The Cox regression model was utilized to analyze the association between the NSAP and the disease-free survival (DFS) of CRC. Predictive performance and clinical utility of prognostic models were evaluated by area under the curve (AUC) and decision curve analyses (DCAs).

**Results:**

During a median follow-up of 23 months, 95 clinical outcomes were observed, with a 1-year survival rate is 89.47%. A comprehensive inflammatory biomarker (NSAP) was established based on four blood indicators (including neutrophil-to-lymphocyte ratio (NLR), neutrophil×monocyte-to-lymphocyte ratio (SIRI), albumin-to-globulin ratio (AGR), and platelet-to-lymphocytes ratio (PLR)). Patients with a lower NSAP had significantly associated with better DFS of CRC (HR=0.53, 95%CI 0.32–0.89). Moreover, compared to a previously established model, the traditional TNM staging system or/and tumor markers, the nomogram based on NSAP displayed more excellent predictive ability (0.752 vs 0.597, 0.711 and 0.735, *P* < 0.05). DCAs also demonstrated that the established nomogram had better utility for decision making.

**Conclusions:**

Our study suggests that NSAP may be a useful comprehensive prognostic biomarker for predicting the DFS of CRC patients. The nomogram based on NSAP can be considered a valuable tool to estimate the prognosis of patients with CRC.

**Supplementary Information:**

The online version contains supplementary material available at 10.1186/s12957-022-02550-0.

## Introduction

Colorectal cancer (CRC) is the third most common malignant tumor and the second leading cause of death worldwide [1]. To date, the risk factors affecting the prognosis of CRC patients have not yet been fully elucidated. Recurrence and metastasis are dismissed as one of the major prognostic factors for CRC patients [2]. After extended radical surgery, patients presenting with recurrence or metastases have 5-year survival rates of less than 33% [3–5]. However, there were limited prediction models were developed to foresee the outcomes of recurrence and metastases. Therefore, it is essential to develop a simple and effective predictive model to provide more research evidence for the prognosis of CRC.

Accumulating studies have shown that the host immune system may have a dual role in tumor promotion and suppression [6–10]. When the body undergoes the immune response and inflammation, the values and ratios of various indicators change in the blood with different degrees, which may be potentially helpful in outcome prediction [11, 12]. Previous studies have revealed that several inflammation-related indicators were independent prognostic factors in the blood for CRC patients, including the neutrophil-to-lymphocyte ratio (NLR), the platelet-to-lymphocyte ratio (PLR), the lymphocyte-to-monocyte ratio (LMR), and the albumin-to-globulin ratio (AGR) [13–16]. However, most of these studies only involve one or several inflammation-related factors and did not systematically explore the combined effects of inflammatory biomarkers in the prognosis of CRC. Moreover, there have been relatively few studies that focus on the outcomes of recurrence and metastasis.

Therefore, the purpose of this study is to comprehensively explore the roles of various blood indicators in the disease-free survival (DFS) of CRC patients and to develop a novel inflammation-related comprehensive biomarker using the LASSO algorithm, with a goal to provide research evidence for individual prediction and decision-making.

## Material and methods

### Study population

From August 2017 to December 2019, a total of 646 CRC patients who underwent surgical resection were consecutively enrolled at Fujian Medical University Union Hospital (Fujian, China). Patients were eligible if they met the following criteria: (1) newly pathologically diagnosed with primary CRC, (2) no receipt of any treatment that affected blood indicators, and (3) without neoadjuvant radiotherapy or radiochemotherapy prior to surgery. The exclusion criteria were as follows: (1) patients with a history of blood disease or infection and autoimmune disease and (2) patients who suffered from other cancers or emergency surgery before being included in the study. All patients provided written informed consent and fully understood the study. The research protocol has been approved by the institutional review board of Fujian Medical University (No.2020KY007) and followed the ethical standards of the Helsinki Declaration.

### Data collection

The patient’s demographic and clinical characteristics were abstracted from the hospital medical record system. The relevant preoperative laboratory parameters for blood indicators were measured on the day of admission, including the tests of blood routine (including neutrophil, lymphocyte, monocyte, high-density lipoprotein counts), blood biochemical (including albumin, globulin counts), and tumor markers including alpha-fetoprotein (AFP), carcinoembryonic antigen (CEA), and carbohydrate antigen 19-9 (CA19-9). Then, eight blood indicators were calculated accordingly: neutrophil-to-lymphocyte ratio (NLR), lymphocyte-to-monocyte ratio (LMR), neutrophil×monocyte-to-lymphocyte ratio (SIRI, Systemic Inflammatory Response Index), albumin-to-globulin ratio (AGR), platelet-to-lymphocyte ratio (PLR), serum albumin + 5×total lymphocyte (OPNI/PNI, Onodera’s Prognostic Nutritional Index), monocyte-to-high-density lipoprotein ratio (MHR), and monocyte-to-lymphocyte ratio (MLR). In addition, we also compared the prognosis of colorectal cancer with a newly developed nomogram in previous literatures [17], including tumor size + NLR + PLR + PNI.

### Follow-up

All patients were followed up by trained study interviewers through telephone inter at 3-month intervals for the first 2 years postoperatively and every 6 months for the next years. DFS was defined as the time from initial surgery to recurrence, metastasis, death (for any reason), or the last follow-up (January 9, 2021). The overall loss of follow-up rate was 4.3%. During follow-up, 28 cases were dead, 82 patients occurred recurrence and metastasis (19 subjects were identified before death), and 551 subjects were still alive at the last follow-up.

### Statistical analysis

The cutoff points for NLR, LMR, SIRI, AGR, PLR, OPNI, MHR, and MLR were determined by the X-tile program (Yale University, Newhaven, Connecticut). Reverse Kaplan-Meier method was used to calculate median follow-up time. A heat map was used to reveal the degree of correlation among the eight blood indicators. Then, the LASSO algorithm was utilized to develop a comprehensive blood indicator. Hazard ratios (HRs) and 95% confidence intervals (CIs) were estimated based on the univariate and multivariate Cox regression model for exploring independent prognostic factors of CRC. Three prediction models were established using a generalized linear model (GLM), receiver operating characteristic (ROC) methods, and the predictive ability that were compared through the area under the curve (AUC). Decision curve analysis (DCA) was plotted to further assess the clinical usefulness of the models. All statistical analysis was performed using R software (version 4.0.5). *P*-value less than 0.05 was statistical significance.

## Results

### Patients’ characteristics

Of the 646 CRC cases included in the final analysis (Table 1), 403 (62.3%) were male patients, and 481 (74.46%) had stage II–III diseases. The majority of patients were diagnosed with adenocarcinoma (87.46%) and moderate tumor differentiation (85.91%). Approximately 10.53–21.12% of patients tended to have elevated or lowered inflammation-related indicators (Table S1). During a median follow-up time of 23 months, a total of 95 patients had outcome events with a 1-year survival rate of 89.47%.

### Establishment of a comprehensive blood index

**Fig. 1:**
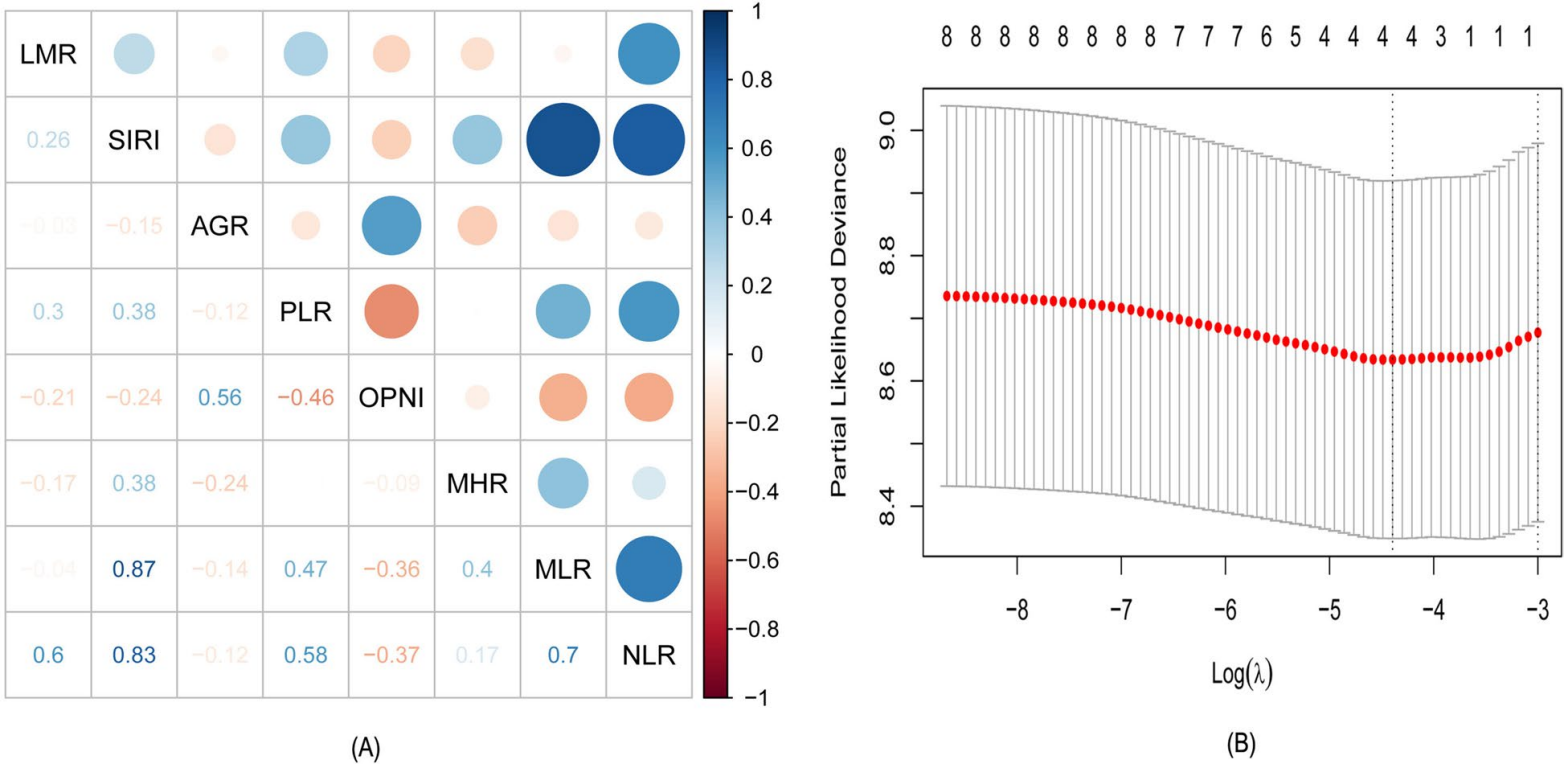
**A** Correlation heat map of inflammation indicators. **B** Partial likelihood deviance of the LASSO analysis

The heat map provided the correlation between eight blood indicators, reflecting in the area of the circle and the shade of the color (Fig. 1A). Several strong correlations were observed between these indicators, among which SIRI and MLR displayed the strongest correlation strength. Given the correlation between the variables and the limitation of the traditional Cox model, the LASSO Cox regression model was used to evaluate the eight selected indicators (Fig. 1B and Fig. S1). The formula for the comprehensive blood index was obtained as follows: NSAP = (0.01567×NLR) + (0.01733×SIRI) + (0.23396×AGR) + (0.00299×PLR). According to this formula, 646 patients were then divided into two groups using the optimal cut-point determined by the X-Tile software: the high-NSAP group (>1.05) and the low-NSAP group (≤1.05).

**Table 1:** Baseline clinicopathological characteristics of patients with colorectal cancer

Variables	No. of patients (%)	No. of outcome (%)
Gender		
Female	243 (37.62)	36 (37.89)
Male	403 (62.38)	59 (62.11)
Age		
≥60	379 (58.67)	53 (55.79)
<60	267 (41.33)	42 (42.21)
Adjuvant therapy		
No	248 (38.39)	28 (29.47)
Chemotherapy	376 (58.20)	63 (66.32)
Other^a^	22 (3.41)	4 (4.21)
BMI		
≥24	198 (30.65)	28 (29.47)
<18.5	40 (6.19)	5 (5.26)
18.5–23.9	408 (63.16)	62 (65.27)
Smoking		
No	602 (93.19)	83 (87.37)
Yes	44 (6.81)	12 (12.63)
Drinking		
No	462 (71.5)	68 (71.58)
Yes	184 (28.5)	27 (28.42)
Comorbidity		
No	310 (47.99)	46 (48.42)
Yes	336 (52.01)	49 (51.58)
Family history of cancer		
No	515 (79.72)	75 (78.95)
Yes	131 (20.28)	20 (21.05)
Tumor stage		
I	120 (18.57)	8 (8.42)
II	233 (36.07)	19 (20.00)
III	248 (38.39)	39 (41.05)
IV	45 (6.97)	29 (30.53)
Specimen type		
Raised type	236 (36.53)	26 (27.37)
Ulcer type	362 (56.04)	52 (54.74)
Other^b^	48 (7.43)	17 (17.89)
Tumor differentiation		
Well	19 (2.94)	3 (3.16)
Moderate	555 (85.91)	73 (76.84)
Poor	33 (5.11)	12 (12.63)
Undifferentiated	39 (6.04)	7 (7.37)
Histological classification		
Adenocarcinoma	565 (87.46)	82 (86.32)
Mucinous carcinoma and other^c^	81 (12.54)	13 (13.68)
Tumor site		
Colon	337 (52.17)	58 (61.05)
Rectum	281 (43.50)	33 (34.74)
Other^d^	28 (4.33)	4 (4.21)
Lymph node metastasis		
No	386 (59.75)	38 (40.00)
Yes	260 (40.25)	57 (60.00)
Tumor size		
≥3cm	460 (71.21)	75 (78.95)
<3cm	186 (28.79)	20 (21.05)

*Abbreviation*: *BMI* body mass index

^a^Included radiotherapy or chemoradiotherapy

^b^Included superficial type, undifferentiated type, and ulcer infiltrating type

^c^Included undifferentiated carcinoma

^d^Included cecum, junction of the rectum and sigmoid colon, and multi-site tumors

**Fig. 2:**
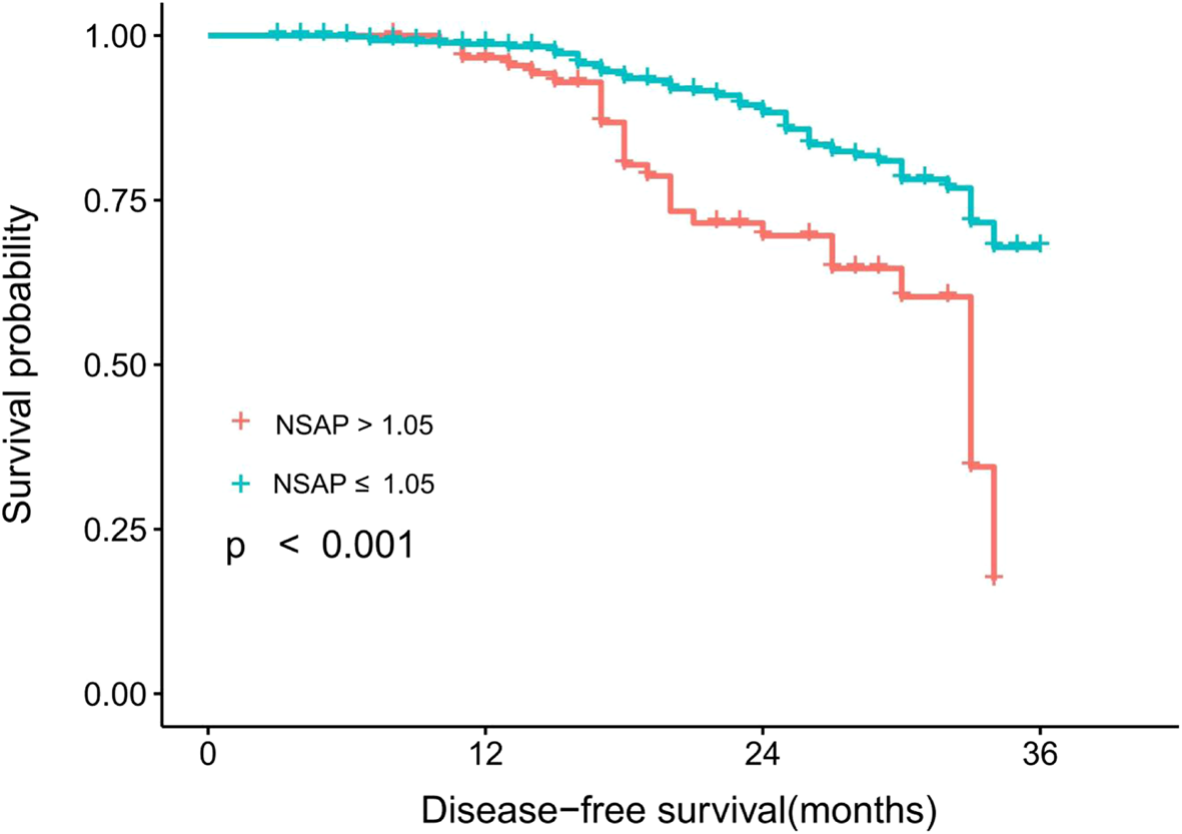
Survival curve of inflammation comprehensive index

The prognostic value of NSAP compared with their higher counterparts, patients with a lower NSAP tended to have a significantly better DFS (*P* < 0.001, Fig. 2). The univariate and multivariate Cox models were both showed that the high-NSAP was associated with an increased risk of DFS: The HRs were 2.56 (95% CI: 1.64-4.00, *P*<0.001), and 1.89 (95% CI: 1.12-3.13, *P*=0.015), respectively. Additionally, tumor stage (IV vs I stage, HR: 4.92, 95% CI 1.62–14.97), AFP (<20 vs ≥20, HR: 0.53, 95% CI 0.33–0.85), and CEA (<5 vs ≥5, HR: 0.53, 95% CI 0.33–0.88) were found to be independent prognostic factors for DFS in the multivariate analysis (Table 2 and Fig. S3).

**Table 2:** Univariate and multivariate analysis for disease-free survival

Variables	Univariate analysis		Multivariate analysis	
	HR (95%CI)	*P*	HR (95%CI)	*P*
Gender (male vs female)	1.01 (0.66–1.52)	0.975	0.97 (0.60–1.57)	0.905
Age (years, <60 vs ≥60)	1.29 (0.86–1.94)	0.213	1.56 (0.97–2.52)	0.069
Adjuvant therapy				
No	Ref.	-	Ref.	-
Chemotherapy	1.49 (0.95–2.32)	0.082	1.12 (0.63–1.98)	0.702
Other	1.70 (0.60–4.86)	0.320	1.12 (0.35–3.57)	0.845
BMI				
≥24	Ref.	-	Ref.	-
<18.5	0.90 (0.35–2.33)	0.828	1.59 (0.58–4.38)	0.368
18.5–23.9	1.09 (0.69–1.70)	0.719	1.23 (0.74–2.02)	0.426
Smoking (yes vs no)	1.11 (0.60–2.05)	0.734	1.09 (0.56–2.11)	0.809
Drinking (yes vs no)	0.96 (0.61–1.50)	0.849	1.16 (0.69–1.93)	0.576
Comorbidity (yes vs no)	1.07 (0.71–1.60)	0.744	1.22 (0.78–1.93)	0.381
Family history of cancer (yes vs no)	0.80 (0.49–1.31)	0.380	0.79 (0.47–1.34)	0.377
Tumor stage				
I	Ref.	-	Ref.	-
II	1.35 (0.59–3.10)	0.473	0.80 (0.32–2.01)	0.641
III	2.60 (1.21–5.58)	0.014	1.04 (0.35–3.13)	0.942
IV	15.70 (7.09–34.56)	<0.001	4.91 (1.61–15.03)	0.005
Specimen type				
Raised type	Ref.	-	Ref.	-
Ulcer type	1.50 (0.94–2.41)	0.090	1.14 (0.68–1.90)	0.619
Other	3.94 (2.14–7.27)	<0.001	1.82 (0.90–3.69)	0.098
Tumor differentiation				
Well	Ref.	-	Ref.	-
Moderate	1.21 (0.38–3.86)	0.747	0.53 (0.15–1.80)	0.306
Poor	4.96 (1.31–16.81)	0.018	1.39 (0.33–5.74)	0.653
Undifferentiated	1.96 (0.50–7.65)	0.333	1.09 (0.25–4.77)	0.911
Histological classification				
Adenocarcinoma	Ref.	-	Ref.	-
Mucinous carcinoma and other	1.22 (0.68–2.19)	0.512	0.48 (0.23–1.03)	0.059
Tumor site				
Colon	Ref.	-	Ref.	-
Rectum	0.70 (0.46–1.07)	0.103	1.07 (0.66–1.73)	0.794
Other	0.85 (0.31–2.34)	0.753	1.19 (0.41–3.47)	0.748
Lymph node metastasis (yes vs no)	2.63 (1.74–3.98)	<0.001	1.93 (0.94–3.95)	0.073
Tumor size (≥3 vs <3)	0.69 (0.42–1.13)	0.144	0.73 (0.42–1.27)	0.261
AFP (<20 vs ≥20)	0.58 (0.37–0.90)	0.016	0.53 (0.33–0.86)	0.010
CEA (<5 vs ≥5)	0.41 (0.27–0.63)	<0.001	0.54 (0.33–0.89)	0.015
CA199 (<37 vs ≥37)	0.40 (0.26–0.62)	<0.001	0.93 (0.55–1.58)	0.786
NSAP (≤1.05 vs >1.05)	0.39 (0.25–0.61)	<0.001	0.53 (0.32–0.87)	0.013

**Fig. 3:**
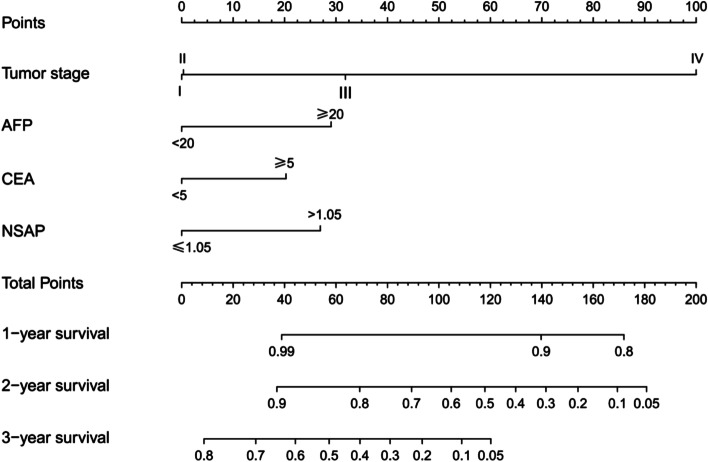
Nomogram composed of tumor stage, AFP, CEA, and NSAP

**Fig. 4:**
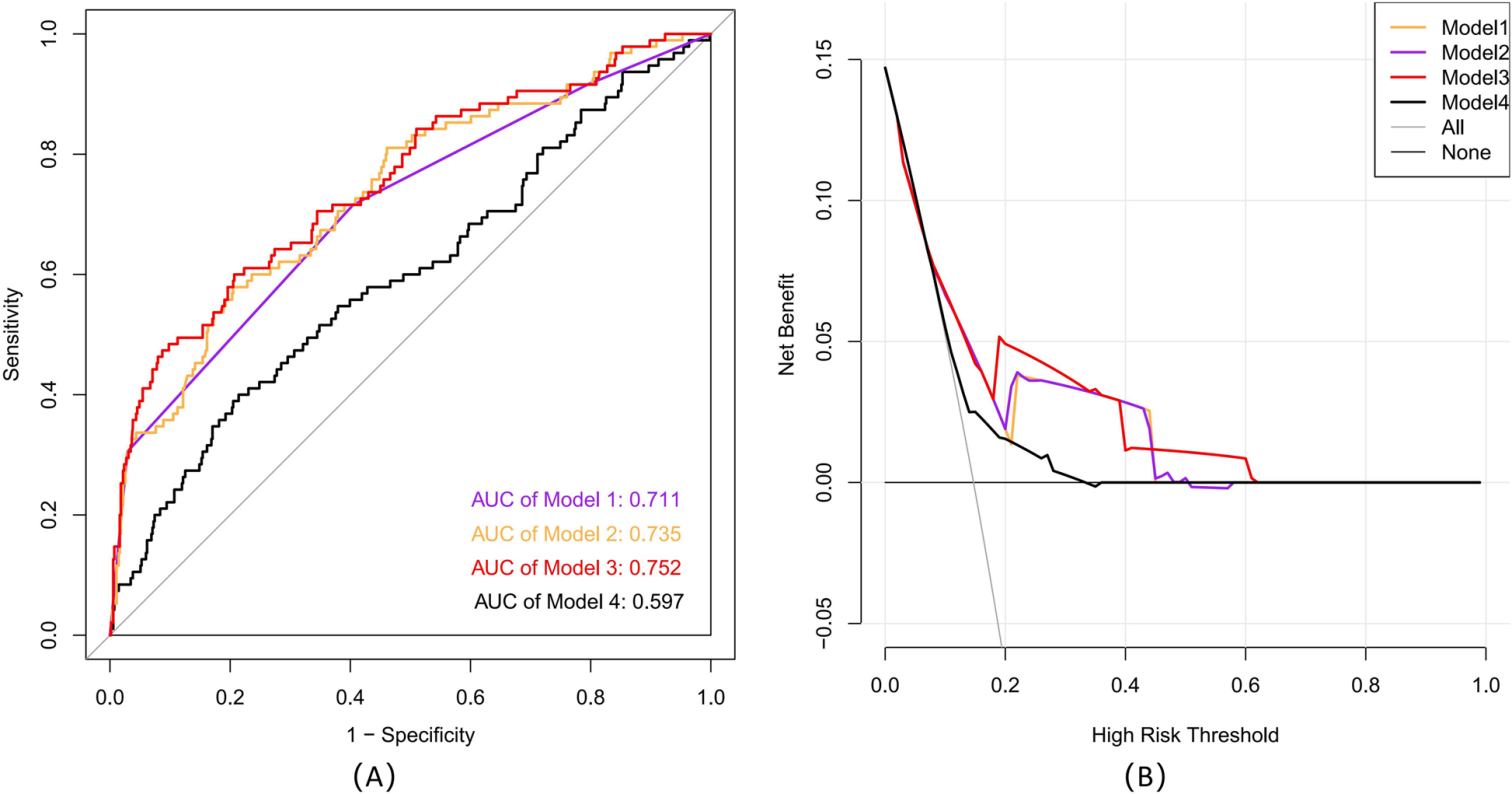
**A** Comparison of AUC between four models. **B** Comparison of DCA between four models

Next, a prognostic nomogram based on the NSAP and other three factors selected by multivariate analysis was developed to quantitatively predict 1-year, 2-year, and 3-year DFS (Fig. 3). The predictive ability of the established nomogram (Model 3) was superior to that of Model 1 (TNM staging system), Model 2 (TNM staging system + AFP + CEA), and Model 4 (tumor size + NLR + PLR + OPNI). The AUC were 0.752 (95% CI 0.717–0.785), 0.711 (95% CI 0.674–0.746), 0.735 (95% CI 0.699–0.768), and 0.592 (95% CI 0.558–0.635), respectively (Fig. 4A). For internal validation, calibration curves for predicting 1-year and 2-year DFS indicated a good match between the predicted probabilities and the actual observations (Fig. S4). In addition, DCA revealed similar results that the established nomogram had more positive net benefit than previously established prognostic nomograms, traditional TNM staging system, or/and tumor biomarkers (Fig. 4B).

## Discussion

To the best of our knowledge, the present study is one of few studies to establish a comprehensive blood index (NSAP) based on a combination of four inflammation-related parameters using the LASSO method. Our study demonstrated that NSAP was an independent prognostic factor for CRC, and the high-NSAP was associated with poor DFS. The nomogram based on NSAP and the TNM staging system and tumor markers displayed a high predictive performance and clinical decision value.

A large number of studies have shown that systemic inflammation has become an essential part of tumorigenesis, proliferation, survival, and migration and is likely to become a new direction for cancer treatment and monitoring [18–20]. To date, several hematological inflammation markers (NLR, OPNI, SIRI, PLR, LMR, AGR) were reported to have potential prognostic values in multiple tumor types [21–26]. In this study, we also found AFP and CEA were valuable independent factors for CRC prognosis, which is supported by results from several previous studies [27, 28]. Of note, except for the above six hands, we also added two additional inflammation indexes (MHR and MLR), which have not yet fully proven the predictive value in the prognosis of CRC. In addition, common prognostic indicators such as lymph node metastasis did not show statistical significance in the multivariate model, which was consistent with a previous study [29]. This may be due to the inclusion of the TNM stage in the multivariate model, resulting in the effect of lymph node metastasis on prognosis may be covered. Additional studies are required to confirm our findings.

Although a single inflammatory index was proved to have predictive ability, the comprehensiveness and accuracy are not satisfactory. In the current study, varying degrees of correlation among the eight indicators were also observed. Therefore, we employed the LASSO method to solve these problems instead of the traditional Cox model. The most prominent advantage of the LASSO algorithm is that the relatively unimportant coefficients of independent variables become 0 and are excluded from modeling through penalized regression on all variable coefficients [30]. It is particularly suitable for linear models with a reduced number of parameters, selection of parameters, and estimation of sparse parameters. Relevant studies have verified that the LASSO model is beneficial to improving the accuracy of the prediction model [30, 31]. In this study, using the LASSO method, we developed a novel comprehensive inflammation index (NSAP), which may be a useful and valuable index for predicting the survival prognosis of patients after CRC operation. In addition, compared with the model established by quoting the article, the model established by us has better predictive performance.

The exact underlying mechanism of the close correlation between the high NSAP and the poor DFS is not yet clear. We found that three of the four indicators included in NSAP are related to lymphocyte count, except for AGR. Previous studies showed that the local control of metastatic invasion by the immune system might be critical to survival. The presence of lymphocytes in the tumor may be a favorable prognostic sign [32]. For example, memory T cells in colorectal cancer can change tumor matrix or tumor cells in the adaptive immune response to reduce the metastatic potential of tumor cells. It may be that the transport characteristics, density, and long-term anti-tumor ability of T cells play a central role in controlling tumor recurrence [33]. Similarly, in solid tumors, tumor-infiltrating lymphocytes show oligoclonal expansion, recognition of tumor antigens, and tumor-specific cytolytic activity in vitro, which are conducive to improving clinical results, including delayed recurrence or delayed death [34, 35]. Additionally , the globulin in AGR, the main protein produced by immune organs, could reflect the body’s inflammation and immune status [36]. Therefore, these indicators have the potential to predict the survival of patients with CRC after surgery.

Nevertheless, the current research has some limitations that should be considered. First of all, although we have collected complete follow-up data of 646 patients, the findings of this study were still limited by the lack of external verification and a relatively small sample size. Second, as a single-center study, it will inevitably bring about potential selection bias. Future multi-center studies with larger sample sizes are warranted to confirm our findings further. Third, the 3-year follow-up time has affected the tracking of more outcome events and limited the observation of the survival rate of CRC for a longer time. It is necessary to continue the follow-up visit according to the established follow-up plan in the future.

## Conclusion

This study developed a novel comprehensive inflammatory biomarker (NSAP) and suggested that NSAP is strongly associated with the DFS in CRC patients. Compared with the previously established model, the traditional TNM staging system, and tumor markers, NASP can effectively improve the predictive ability of the prognostic model. More prospective multi-center data sets are still needed to confirm our findings and the practicality of clinical decision-making in the future.

## Supplementary Information

**Additional file 1: Table S1.** Baseline tumor markers and inflammation index of patients with colorectal cancer. **Fig.** **S1.** Coefficients deviance of the LASSO analysis. **Fig. S2.** A, B, C X-tile analysis on the optimal cutoff points of NSAP. **Fig. S3.** A Kaplan-Meier curves for disease-free survival of CRC patients. according to the stage; B according to alpha-fetoprotein (AFP); C according to carcinoembryonic antigen (CEA). **Fig. S4.** A Calibration curve of nomogram of 1-Year DFS. B Calibration curve of nomogram of 2-Year DFS.

## Data Availability

The datasets used and/or analyzed during the current study are available from the corresponding author upon reasonable request.

## Abbreviations

CRC: Colorectal cancer
LASSO: The least absolute shrinkage and selection operator method
NSAP: A comprehensive biomarker
DFS: The disease-free survival
AUC: Area under the curve
DCAs: Decision curve analyses
AFP: Alpha-fetoprotein
CEA: Carcinoembryonic antigen
CA19-9: Carbohydrate antigen 19-9
NLR: Neutrophil-to-lymphocyte ratio
LMR: Lymphocyte-to-monocyte ratio
SIRI: Systemic Inflammatory Response Index
AGR: Albumin-to-globulin ratio
PLR: Platelet-to-lymphocyte ratio
OPNI/PNI: Onodera’s Prognostic Nutritional Index
MHR: Monocyte-to-high-density lipoprotein ratio
MLR: Monocyte-to-lymphocyte ratio
HRs: Hazard ratios
CIs: Confidence intervals
GLM: Generalized linear model
ROC: Receiver operating characteristic
