# Supplementary material for: A novel inflammation-related prognostic biomarker for predicting the disease-free survival of patients with colorectal cancer
Source: World J Surg Oncol. 2022 Mar 11;20:79. doi: 10.1186/s12957-022-02550-0 (PMC8917685; doi:10.1186/s12957-022-02550-0)
Supplement: Supplementary file 1 — Additional file 1: Table S1. Baseline tumor markers and inflammation index of patients with colorectal cancer. Fig. S1. Coefficients deviance of the LASSO analysis. Fig. S2. A, B, C X-tile analysis on the optimal cutoff points of NSAP. Fig. S3. A Kaplan-Meier curves for disease-free survival of CRC patients. according to the stage; B according to alpha-fetoprotein (AFP); C according to carcinoembryonic antigen (CEA). Fig. S4. A Calibration curve of nomogram of 1-Year DFS. B Calibration curve of nomogram of 2-Year DFS. [file 12957_2022_2550_MOESM1_ESM.docx]

Supporting Material

# Tables and Figures

## Tables

| Table S1 Baseline tumor markers and inflammation index of patients with colorectal cancer | | |
| --- | --- | --- |
|  |  |  |
| Variables | No. of patients (%) | No. of outcome (%) |
| AFP |  |  |
| ≥20 | 5 (0.77) | 1 (1.05) |
| <20 | 641 (99.23) | 94 (98.95) |
| CEA |  |  |
| ≥5 | 261 (40.40) | 31 (32.63) |
| <5 | 385 (59.60) | 64 (67.37) |
| CA19-9 |  |  |
| ≥37 | 88 (13.62) | 8 (8.42) |
| <37 | 558 (86.38) | 87 (91.58) |
| NLR |  |  |
| ≥2.88 | 130 (20.12) | 31 (32.63) |
| <2.88 | 516 (79.88) | 64 (67.37) |
| LMR |  |  |
| ≥12.24 | 110 (17.03) | 20 (21.05) |
| <12.24 | 536 (82.97) | 75 (78.95) |
| SIRI |  |  |
| ≥0.34 | 575 (89.01) | 81 (85.26) |
| <0.34 | 71 (10.99) | 14 (14.74) |
| AGR |  |  |
| ≥1.12 | 574 (88.85) | 87 (91.58) |
| <1.12 | 72 (11.15) | 8 (8.42) |
| PLR |  |  |
| ≥233.08 | 68 (10.53) | 21 (22.11) |
| <233.08 | 578 (89.47) | 74 (77.89) |
| OPNI |  |  |
| ≥56.50 | 74 (11.46) | 7 (7.37) |
| <56.50 | 572 (88.54) | 88 (92.63) |
| MHR |  |  |
| ≥0.51 | 122 (18.89) | 24 (25.26) |
| <0.51 | 524 (81.11) | 71 (74.74) |
| MLR |  |  |
| ≥0.33 | 95 (14.71) | 25 (26.32) |
| <0.33 | 551 (85.29) | 70 (73.68) |
| Abbreviation: AFP, alpha fetoprotein; CEA, carcinoembryonic antigen; CA19-9 cancer antigen 19-9; NLR, neutrophil count/lymphocyte count; LMR, lymphocyte count/monocyte count; SIRI (Systemic Inflammatory Response Index), neutrophil count × monocyte count/lymphocyte count; AGR albumin count/globulin count, PLR platelets count/lymphocytes count; OPNI (prognostic nutritional index), serum albumin count + 5 × total lymphocyte count; MHR, monocyte count/high density lipoprotein count; MLR, monocyte count/lymphocyte count. | | |

## Figures


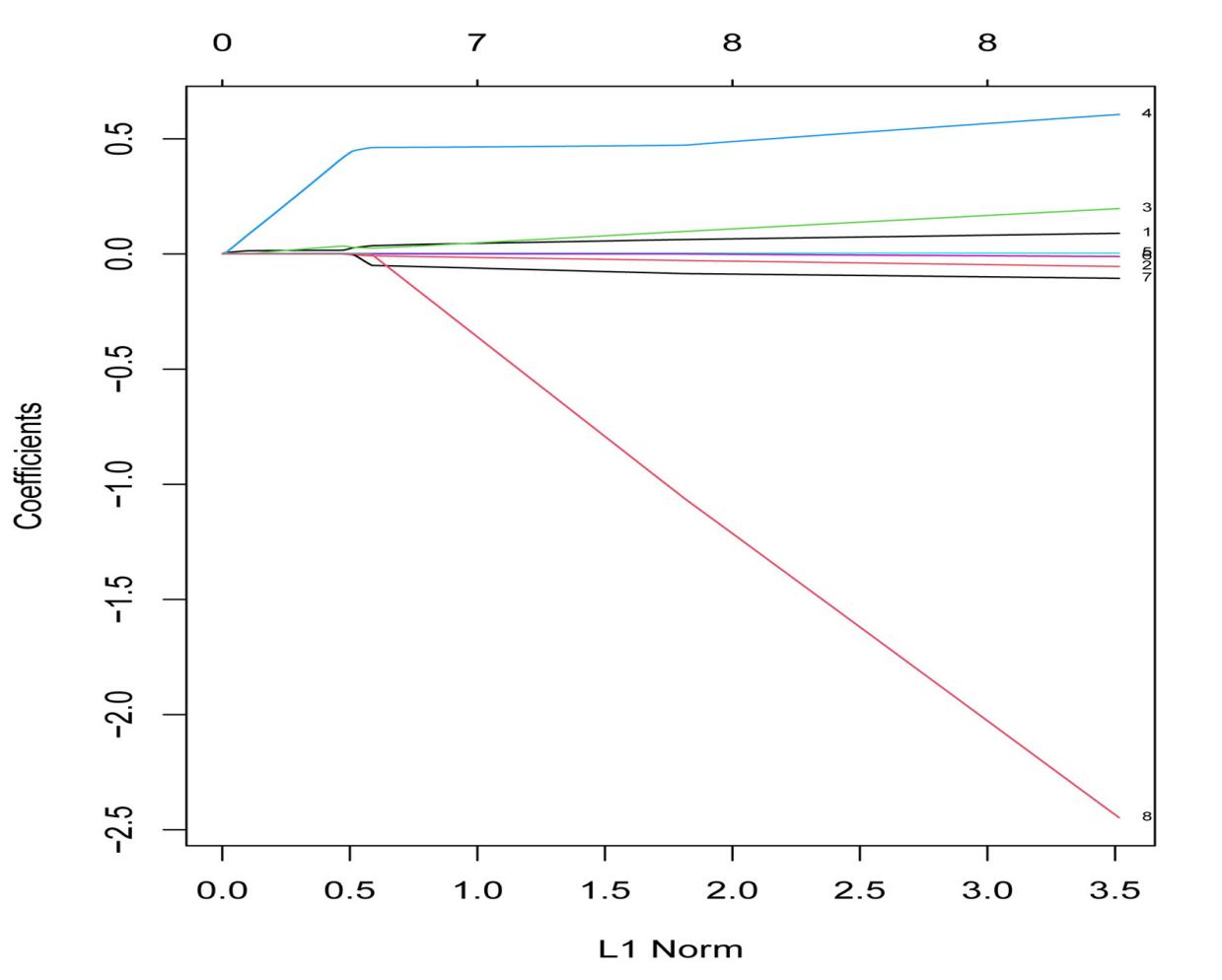


## Fig. S1 Coefficients deviance of the LASSO analysis.


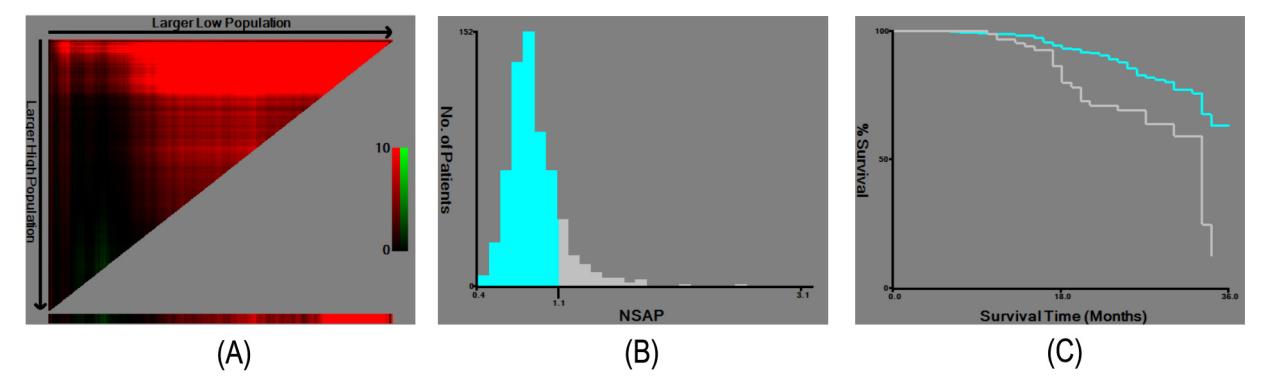


**Fig. S2** A, B, C X-tile analysis on the optimal cutoff points of NSAP.

**
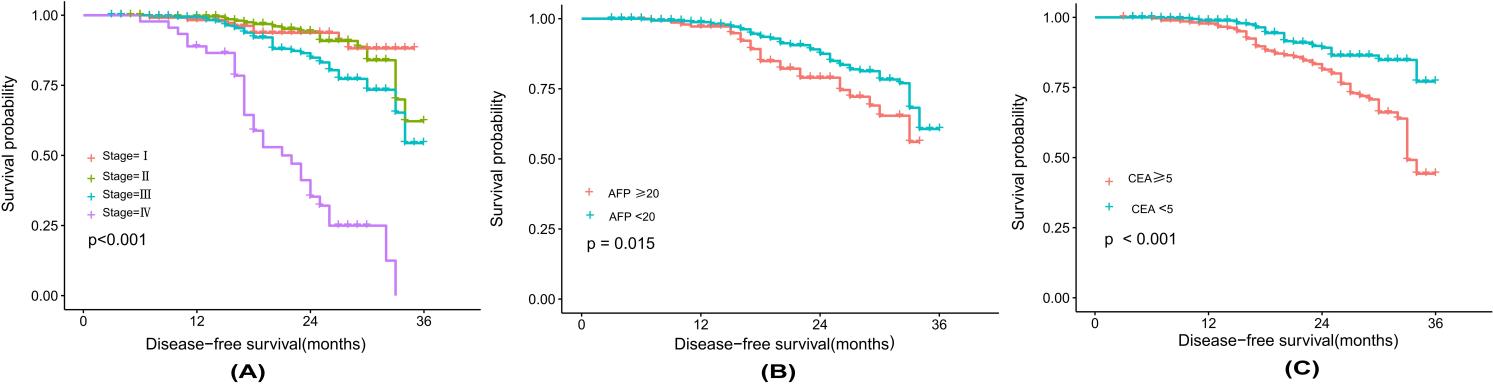
**

**Fig. S3** A Kaplan-Meier curves for disease-free survival of CRC patients. according to stage; B according to alpha-fetoprotein (AFP); C according to carcinoembryonic antigen (CEA).


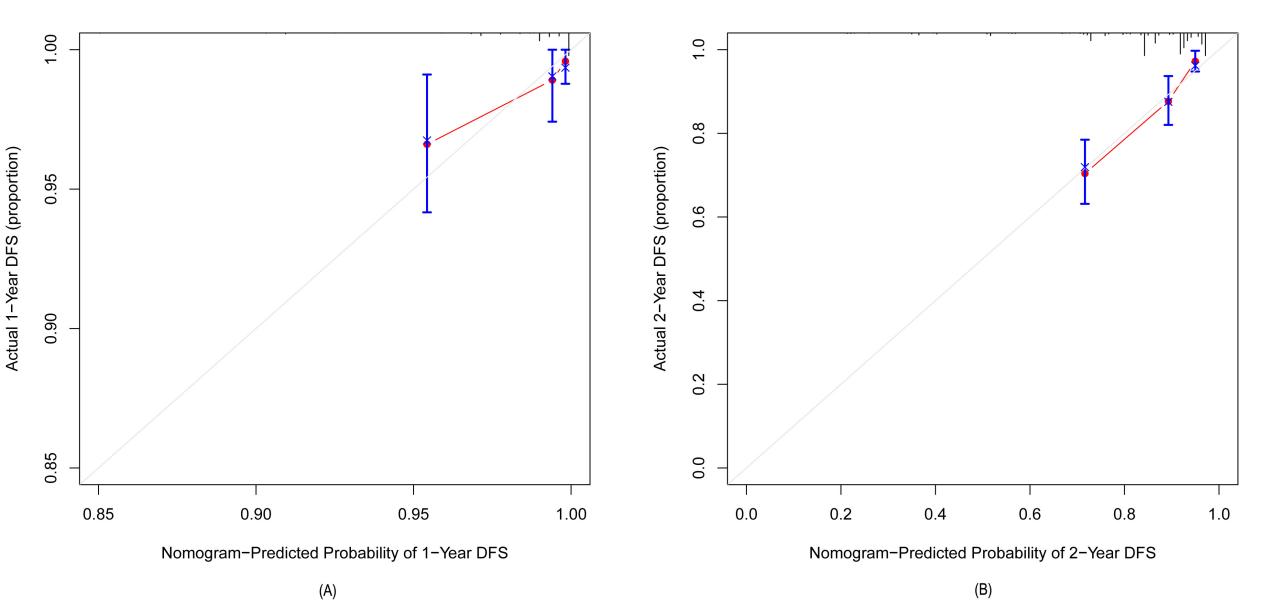


**Fig. S4** A Calibration curve of nomogram of 1-Year DFS. B Calibration curve of nomogram of 2-Year DFS.
